# A systematic review of interventions to increase attendance at health and fitness venues: identifying key behaviour change techniques

**DOI:** 10.1186/s12889-020-09898-6

**Published:** 2020-12-07

**Authors:** Matthew Rand, Paul Norman, Elizabeth Goyder

**Affiliations:** 1 School of Health and Related Research, The University of Sheffield, 30 Regent St, Sheffield, S1 4DA UK; 2 Department of Psychology, The University of Sheffield, Cathedral Court, The University of Sheffield, 1 Vicar Ln, Sheffield, S1 2LT UK

**Keywords:** Physical activity, Public health, Health and fitness, Interventions, Attendance, Behaviour change

## Abstract

**Background:**

Members’ attendance at health and fitness venues typically declines over the course of their membership, with a likely negative impact on physical activity and health outcomes. This systematic review sought to examine the effectiveness of interventions to increase attendance at health and fitness venues and identify the behaviour change techniques (BCTs) included in effective interventions.

**Methods:**

A systematic search of seven databases was conducted. The Behaviour Change Technique Taxonomy was used to code the interventions. Cohen’s *d* was used to assess the effectiveness of the interventions.

**Results:**

Fourteen papers reporting 20 interventions were included in the review. Most interventions were found to have trivial or small effects on attendance, although one had a medium effect (*d* = 0.60) and three had a large effect (*d*s = 1.00, 1.37, 1.45). The interventions used a limited range of BCTs, with “Prompts/Cues” being the most frequently used. Of the interventions with large effect sizes, two used “Problem solving” and “Pros and cons” and one used “Goal setting (behaviour)” and “Review behaviour goals”.

**Conclusions:**

Only a small number of studies have tested interventions to increase attendance at health and fitness venues, with predominantly trivial or small effects. With the possible exception of problem solving alongside decisional balance and goal setting alongside reviewing behaviour goals, there is little evidence for the effectiveness of specific BCTs. Further research is required to identify the key components of effective interventions to increase attendance at health and fitness venues.

## Background

Worldwide, it is estimated that 31% of adults aged 15 and over are inactive; that is, they do not meet the recommended guidance of 150 min of moderate-intensity aerobic physical activity (PA), or at least 75 min of vigorous-intensity aerobic PA, per week [1]. Thus, there is a clear need to increase PA in a significant proportion of the population. Public Health England (PHE) has identified a range of sectors that are well positioned to help the population become more active including local and national government, schools, health services, the transport sector, voluntary organisations and the sport and leisure sector [2]. Of these, the sport and leisure sector is the only one to provide PA as a direct service and is therefore well placed to support increases in PA levels. However, to date, there is limited evidence about how this sector can increase PA levels in the population [3]. Health and fitness is a large subsector of the sport and leisure industry, with approximately 60 million people in Europe having membership of a health and fitness organisation which gives them access to a venue [4]. Within the UK, approximately 15% of the population are estimated to be members of a health and fitness organisation [5]. Health and fitness venues typically provide PA equipment that can be used within gyms, they offer exercise classes led by trained instructors and offer swimming pool provision. Individuals typically pay a membership fee to use these facilities. Given that health and fitness organisations provide venues and activities that have the potential to increase PA levels in the population, and that many individuals primarily subscribe to use health and fitness facilities for health reasons (e.g., to lose weight, for increased fitness) [6], they provide an ideal context in which to study initiatives to increase PA levels.

Despite the level of health and fitness membership, attendances at health and fitness venues generally decline from the start of an individuals’ membership [7]. Moreover, many members do not use their membership [8]. A recent study in the UK found that only 22% of new members attended a health and fitness venue 12 months after the start of their membership [7]. A study in the United States also reported a mean attendance of approximately four times a year for members on an annual contract [8]. It is likely that many of these members are not meeting recommended PA guidelines, given that most members join for health reasons [6]. Therefore, interventions that increase attendance at health and fitness venues are also likely to have a positive impact on public health.

To identify the most effective interventions to increase attendances at health and fitness venues, it is important to understand which interventions have previously been tried, the extent to which they have influenced attendance behaviour, and the intervention components that were key to behaviour change. Such research can provide useful information for health and fitness organisations about where to place their resources to increase member attendances at their venues. Such information would also be useful for national policy makers and global organisations such as the WHO to help inform future recommendations for promoting PA [9] (e.g., ‘What Works’ guidance). To date, very little is known about the effectiveness of interventions to increase attendance at health and fitness venues; the current review aims to fill this gap.

Coding a behaviour change technique (BCT), defined as an observable and replicable component of an intervention designed to alter processes that regulate behaviour within an intervention [10], can help to identify the key techniques, or “active ingredients”, of an intervention. Understanding interventions that are effective in promoting behaviour change requires clear reporting and a standard for outlining the content and descriptions of interventions [11]. Thus, the current review utilised the 93 BCT taxonomy (v1) [10] to code interventions that have attempted to increase attendances at health and fitness venues. Effective BCTs have been identified for promoting PA in general [12]. However, to date, there has been no research investigating the BCTs used in interventions aimed to increase attendance at health and fitness venues. The BCTs that help to increase PA may or may not be the same as those that are important in increasing attendance at health and fitness venues.

This systematic review therefore aimed to: 1) assess the effectiveness of interventions designed to increase attendance at health and fitness venues; 2) identify the BCTs that have been used in interventions to increase attendance at health and fitness venues; and 3) assess the relative effectiveness of different BCTs used to increase attendance at health and fitness venues.

## Method

### Search strategy, selection criteria and data extraction

Relevant health, psychological and exercise related electronic databases were selected; Business Source Premier, Cochrane Controlled Trials Register, Google Scholar, MEDLINE, Physical Education Index, PsychINFO and Scopus. Searches were carried out in June 2019. Only English language reports were included. There was no restriction on publication date. Reference lists and citations of identified studies were also scanned. Grey literature, including conference proceedings and abstracts were searched to identify research that may have been presented ahead of full publication. Only studies that tested interventions to increase attendance behaviour in a health and fitness venue using a randomised controlled experimental design were included in the review. Randomised controlled trials (RCTs) are considered to be the ‘gold standard’ design to provide evidence of effectiveness of an intervention and minimise the risk of bias [13]. Other designs such as nonrandomised or observational studies were excluded. Studies located in a health and fitness venue with adult members of the venue were included. Studies involving non-members or volunteers were excluded, as were studies involving participants who were suffering with a clinical condition or were part of an exercise referral scheme. Studies which only measured attendance at specific exercise sessions or programmes were excluded. The first author retrieved data, which was checked by the second author, from the included studies and recorded these on a standardised data extraction form. The following details were retrieved: author and country; sample; setting; conditions; BCTs; attendance measure; main findings; and effect size as assessed by Cohen’s *d* [14]. In line with Cohen’s guidelines [14], *d* < 0.20 was interpreted as trivial, *d* ≥ 0.20 was interpreted as a small effect size, *d* ≥ 0.50 as a medium effect size, and *d* ≥ 0.80 as a large effect size. The conditions were coded such that a positive effect size would indicate a positive effect of the intervention on attendance relative to the control condition.

### Quality of the included studies

The Cochrane tool to assess risk of bias in RCTs [15] was used to assess the quality of the included studies. This considers bias in terms of *selection, performance, detection, attrition, reporting* and *other biases* and studies are rated as high, low or unclear in the risk of bias for each domain. These criteria were used to rate each of the included studies.

### Behaviour change techniques (BCTs)

The BCTs used in each study were identified from intervention descriptions and coded from the BCT taxonomy (v1) according to the instructions provided. BCTs in the intervention condition were coded. Where it was not possible to code an intervention component to one of the 93 BCTs as described in the taxonomy, additional techniques were coded and named as appropriate.

## Results

### Included studies

**Fig. 1:**
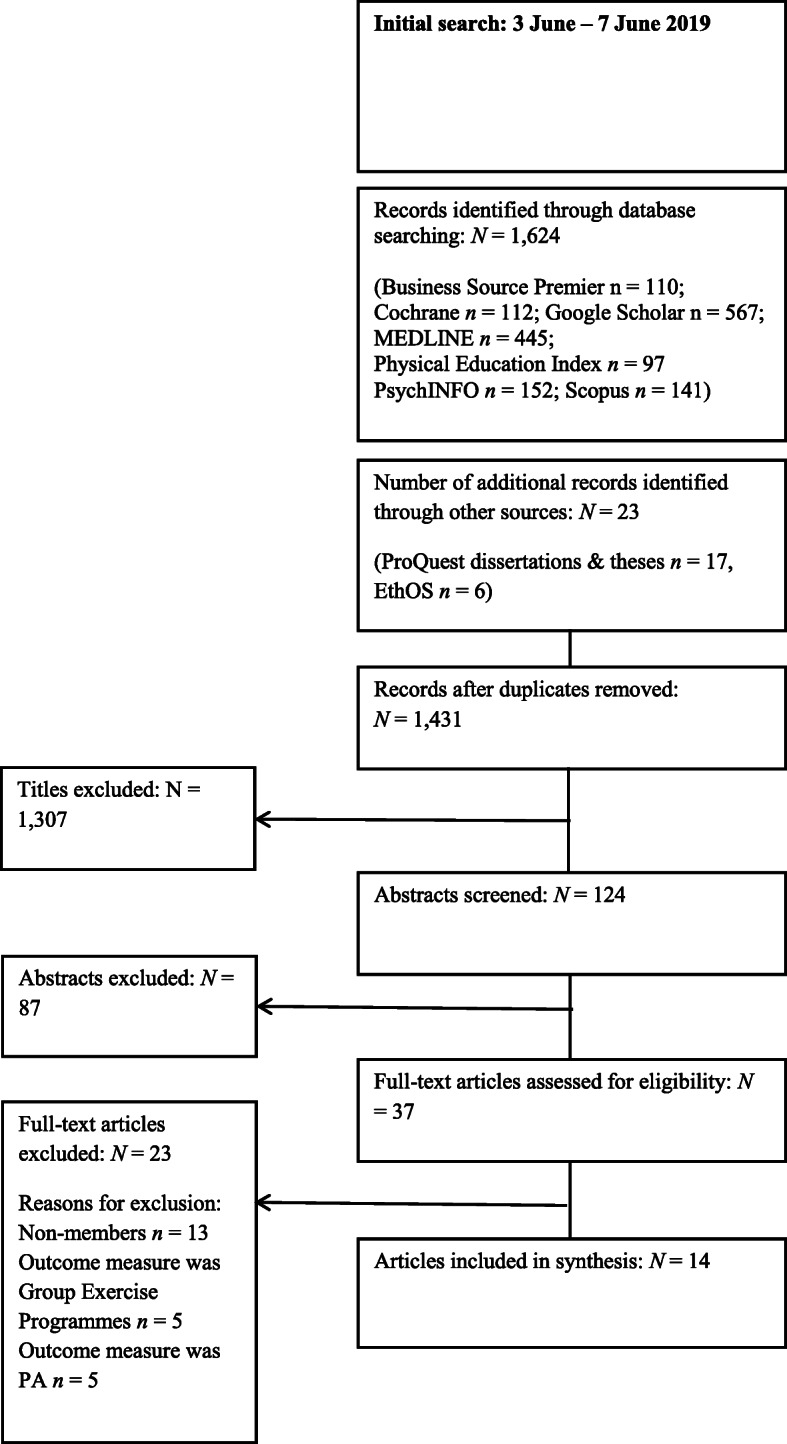
PRISMA Flow Diagram Showing Study Selection Process

Fourteen studies, including 20 interventions, were identified that met the inclusion criteria (see Fig. 1). The publication dates ranged from 1977 [16] to 2018 [17, 18]. A description of the included studies is presented in Table 1.

**Table 1:** Study Characteristics

Author and country	Participant Characteristics	Intervention length, content and groups	Measures	Recorded Results [actual *p* values reported where identified by authors]	Effect Size *d*
**Annesi (2002)** [19] **Italy**	*N* = 100 [gym members] Intervention condition *N* = 50 Control condition *N* = 50	All participants were told that completing three sessions (or more) of vigorous exercise per week was recommended for fitness progress. All participants were provided individual appointments of 40 min with the same exercise professional every 6 weeks Control condition’s meetings focused on the transfer of physiological knowledge, the need to continue exercise having positive effects on health and personalised modification of exercise plans on progress Intervention condition had an additional focus which was the implementation of a goal-setting protocol	Attendance was calculated for the 52 weeks of the intervention	Over the study period there was greater attendance in the intervention condition than the control condition (*p* < .0001)	1.37
**Calzolari et al. (2017)** [20] **Italy**	*N* = 247 [university students] Intervention condition *N* = 89 Control condition *N* = 158	Intervention condition received weekly emails reminding them of the opportunity to attend the gym (during a maximum period September 1, 2009 to March 16, 2010). Control condition did not receive reminders	Attendance was monitored during and two years after the treatment period	The intervention condition had 0.6 more visits per month than the control condition during the treatment period but this was not significant [*p* > 0.05] There was no significant difference in the number of visits per month between the intervention and control conditions at two year follow-up [*p* > 0.05]	0.16 0.08
**Carrera et al. (2017)** **USA** [21]	*N* = 690 [new gym members] Intervention condition *N* = 514 Control condition *N* = 176	Intervention condition received one of three incentives if they attended the gym at least 9 times over the first 6 weeks of their membership; a 30 dollar payment (“money30”), or a 60 dollar payment (“money60”) or an item they had chosen costing 30 dollars (“item”) Control condition received 30 dollars payment unconditionally during the same period	Attendance was monitored for the first 12 weeks of the members’ gym membership (including the six week intervention period at the beginning of their membership)	For the intervention condition as a whole, incentives did not have a statistically significant impact on attendance during the first six weeks [*p* > 0.05 0.10]	0.08
**Carrera et al (2018)** **USA** [18]	*N* = 877 [members of a private gym] Intervention condition *N* = 438 Control condition *N* = 439	Intervention condition were asked to check off the time they planned to work out that day each day in a two week period [participants were told that the information would be used to create calendar invitations for each day/time they planned to visit] Control condition were asked to check off a time that they worked out in the preceding two weeks	Attendance was monitored between the two conditions during the experimental period	There was no significant difference between the intervention and control conditions during the experimental period [*p* > 0.05]	0.10
**Courneya et al (1997)** [22] **Canada**	*N* = 300 [alumni, support staff, academic staff, and the general public. University students were excluded] Intervention condition *N* = 100 Active control condition *N* = 100 Control condition *N* = 100	Intervention condition participants received a letter by mail, containing a friendly message and pamphlet outlining the possible activities available at the fitness facility. The letter included an additional paragraph instructing them that they could earn one month’s free membership if they attended the fitness facility at least 12 times in the next month Active control condition participants received the same letter by mail as the intervention condition, without the additional paragraph instructing them that they could earn 1 month’s free membership if they attended the fitness facility at least 12 times in the next month The control condition participants received no intervention	Attendance of all participants was monitored for one month following the intervention	The intervention condition had significantly higher attendance than the active control condition over the one month period [t(198) = 2.76, *p* < 0.05] The intervention condition did not have significantly higher attendance than the control condition [*p* > 0.05]	0.38 0.14
**Estabrooks et al (1996)** **Canada** [23]	*N* = 200 [Alumni, support staff, academic staff, and the general public. University students were excluded] Intervention condition *N* = 100 Active control condition *N* = 50 Control condition *N* = 50	Intervention condition participants received a letter by mail, which contained a friendly message and outlined the possible activities at the fitness facility. They also received a key chain that was to act as a stimulus control and a brief statement about the purpose of the key chain. At the completion of the 8 week observation period, they received a telephone call as a manipulation check Active control condition received a letter by mail, which contained a friendly message and outlined the possible activities at the fitness facility. They did not receive the additional stimulus The control condition participants received no intervention	Attendance was monitored for eight weeks following the intervention	There was no main effect for the intervention condition [*F* (197) = .47, *p* > 0.05]	0.05
**Marz (2017)** [24] **Germany**	*N* = 94 [registered members of the gym] Intervention condition *N* = 60 Control condition *N* = 34	Intervention condition participants were split into a “gain-treatment” or “loss-treatment”. In the “gain-treatment”, participants were rewarded for frequent attendance at the gym. In the “loss-treatment”, incentives were framed in a way that infrequent attendance at the gym was penalized Control condition participants received no financial incentives	Attendance was monitored for the four week intervention and 12 weeks after the intervention	Participants assigned to the “loss- treatment” had an estimated average of 0.686 additional visits per week in the intervention period compared to the control condition, which was statistically significant [*p* < 0.05] Participants assigned to the “gain-treatment” had an estimated average of 0.344 additional visits per week compared to the control condition, which was not statistically significant [*p* > 0.05]	0.33 0.23
**Muller and Habla (2018)** [17] **Sweden**	*N* = 2463 [new registered members of the gym] Intervention condition *N* = 1231 Control condition *N* = 1232	Intervention condition received a series of email reminders over the course of a 3 month period [January 9, 2017 and April 9, 2017] encouraging them to attend the gym. The control condition received no email reminders	Attendance data was analysed during the intervention period	During the intervention period, the intervention condition had a slightly higher attendance than the control condition [total visits increase by 13%] (*p* < 0.01)	0.01
**Nigg et al (1997)a** [25] **Canada**	*N* = 204 [Alumni, support staff, academic staff, and the general public] Intervention condition *N* = 154 Control condition *N* = 50	The three experimental conditions received a letter by mail that contained a friendly message and a calendar month with large squares containing four weeks beginning November 13 and ending December 10. Participants were unaware that the study focused on the motivational effects of self-monitoring or that their attendance was being objectively monitored by the researchers. Participants in the “Positive SM” condition were instructed to place an “X” in each calendar day they attended the fitness facility. Participants in the “Negative SM” condition were asked to place an “X” in each calendar day that they did not attend the fitness facility. Participants in the “Neutral SM” were instructed to place a “tick” in each calendar day that they attended the facility and a “X” in each day they did not attend the facility. The control condition received no intervention	Attendance was monitored for four weeks post intervention	The “Positive SM” condition showed a significantly higher attendance than the control condition post-intervention (p < 0.05) The “Negative SM” condition showed a significantly higher attendance than the control condition post-intervention (p < 0.05) The “Neutral SM” condition showed a non-significant difference in attendance post intervention compared to the control condition (*p* > 0.05)	*d* = 0.08 0.20 0.02
**Nigg et al (1997)b** [26] **Canada**	*N* = 153 [Alumni, support staff, academic staff, and the general public] Intervention condition *N* = 102 Control condition *N* = 51	Intervention condition participants received a telephone call ‘interview’ in which they were asked to think systematically of and record the expected gains and losses of either exercising at the gym (relevant scenario) or not smoking (irrelevant scenario) Control condition participants received no intervention	Attendance was monitored for four weeks of baseline and the eight weeks of the intervention The number and importance of pros and cons listed by each individual in the relevant DBS condition was examined	Attendance in the relevant DBS condition saw virtually no change from baseline to the end of the intervention [t(50) = .26, *p* > 0.05] while attendance in the control condition saw a significant decrease from baseline to the end of the intervention f(50) = 1.94. *p* < .03.	0.31
**Rohde et al. (2017)** [27] **Netherlands**	*N* = 1182 [members of the gym] Intervention condition *N* = 258 (Unconditional rebate *n* = 48; Conditional rebate *n* = 113; Choice *n* = 97) Control condition *N* = 924	Intervention condition participants were randomly split into ‘conditional’, ‘unconditional’ or ‘choice’ conditions. The ‘conditional’ participants received a rebate of approximately 10% of the average membership fee conditional on attending the gym at least once per week in 11 of the 13 weeks of the first quarter in 2010. This incentive was repeated in the following quarter. The ‘unconditional’ condition participants received the 10% rebate per quarter for staying a member of the gym. The ‘choice’ participants could choose between the conditional or unconditional rebate. Control condition participants did not receive any incentives	Attendance of participants was monitored for 15 months in total; the quarter before the intervention, the two quarters of the intervention and the two quarters following the intervention	The only increase in attendances during the intervention period was for the conditional rebate (CR) and unconditional rebate (UR) conditions in the first quarter of 2010. There was no effect when comparing each of the intervention conditions to the control condition (*p* > 0.05).	[UR: *d* = − 0.03] [CR: *d* = − 0.004]
**Spangenberg (1997)** [28] **USA**	*N* = 142 [members of the club] Intervention condition *N* = 73 Control condition *N* = 69	Intervention condition participants received a telephone call asking whether they were a member of a health club and then asked “Do you expect to use the club in the next week?” Control condition participants received the same telephone call as the intervention condition, but were not asked the question “Do you expect to use the club in the next week?”	Attendance was monitored for the 10 day period immediately following telephone contact and for the six-month period following the intervention	Over the ten day period, 12% of the intervention condition participants and 7% of the control condition participants attended the club once or more during the ten day period, however this was not statistically significant (χ^2^ = 1.12, *df* = 1, *p* > 0.05) For the six month period, the average number of visits was 10.25 for the intervention condition which was double the control condition average of 5.1 visits. This was significant at the 5% confidence level (F(l,93) = 3.78, *p* = 0.05).	0.18 0.10
**Thompson et al (1980)** [29] **Canada**	*N* = 36 [adult female members of the gym] Intervention condition *N* = 18 Control condition *N* = 18	All participants were contacted by telephone to arrange a meeting for a new exercise programme offered by the club. Participants at this initial meeting were asked to complete a series of personal inventories and to express their relative preferences for a number of exercises Participants were then randomly assigned to the treatment conditions and returned for a second visit to the club Intervention condition participants were told that their programme was based totally on the choices they had made. At the end of the second visit they were asked to select six additional exercises which they would add to their programme – one every third visit Control condition participants were told that their programme was based on a standardised exercise format rather than on their expressed preferences. At the end of their second visit they were told that six additional exercises would be added to their programmes by the instructors	Attendance was monitored over a six week period following the intervention	The intervention condition had a higher average attendance than the control condition over the 6-week period, however, this was not statistically significant [F(1,34) = 2.88, *p* > 0.05]	0.29
**Wankel et al (1977)** [16] **Canada**	*N* = 100 [adult female members of the gym] Intervention condition = 75 [‘Complete decision’ *n* = 25, ‘Positive-only’ *n* = 25, ‘Regular call up’ *n* = 25, Control condition *n* = 25	The ‘complete decision-balance-sheet’ treatment received a telephone call where they were asked to complete a decision balance sheet grid concerning attendance of the health club’s programmes. The ‘positive-only’ telephone interview condition were only asked to think of and report positive outcomes to be expected. A further condition (‘Regular call up ‘received a standard telephone call utilised by the club in following up inactive members. This call attempted to establish why members had not been utilising their membership and encouraged them to use it more in the future. This condition served as a “personal attention” control condition for the other two intervention conditions The control condition received no intervention	Attendance was monitored for one month following the intervention	The three treatment conditions had a significantly higher attendance than the control condition (*p* < 0.05) The “Positive only” condition had the highest attendance compared to the control condition. This was followed by the ‘complete decision-balance-sheet’ condition. The regular call up condition had the smallest attendance difference from the control group	1.45 1.00 0.60

### Participants

A total of 6788 participants were included in the 14 studies, with 3406 randomised to receive an intervention and 3382 to a control condition. The number of participants completing the studies (intervention and control) ranged from 36 [29] to 2463 [17]. The mean age of the participants ranged from 28 [29] to 41 [19] years old, with half of the studies reporting a mean age within the thirties. Twelve of the 14 studies included both females and males in their interventions. The remaining two studies only included females [16, 29]. Other demographics such as education, ethnicity and employment status were inconsistently reported.

### Mode of delivery

The mode of delivery varied in the studies. There were two main modes of delivery; one study included multiple meetings with participants and was primarily face-to-face [19], the remaining studies used methods which were not face-to-face (e.g., email reminders, telephone calls, letters).

### Length of intervention and monitoring periods

Seven interventions were one-off interventions (e.g., a letter in the post) [16, 22, 23, 25, 26, 28, 29] and the remaining seven interventions [17–21, 24, 27] varied between two weeks [18] and two years [19] in length. The median length was 12 weeks. All of the studies measured attendance either during the intervention and/or for a period of time after the intervention had taken place. The monitoring period of attendance ranged from two weeks [18] to two years [20]. The median monitoring period was 8 weeks.

### Outcome measures

All 14 studies reported objective, electronically recorded attendance at the health and fitness venue.

### Quality of the included studies

**Fig. 2:**
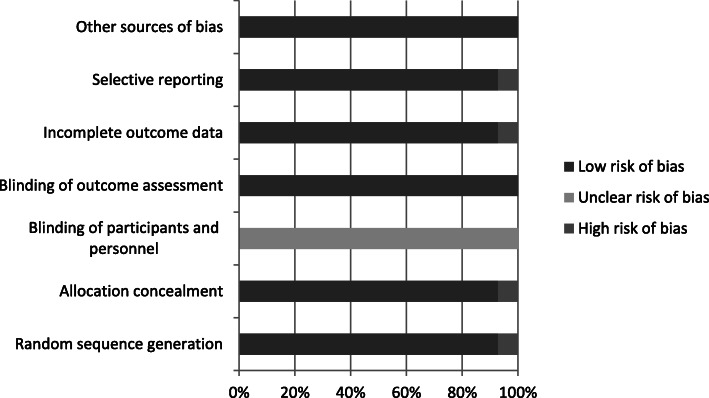
Bias Chart for Included Studies

Overall, the included studies reported a low level of bias within the assessment. Bias was reported to be high once each in “random sequence generation” [20], “allocation concealment” [20], “selective reporting” [20] and “incomplete outcome data” [24]. A summary analysis of the level of bias in each of the included studies is presented in Fig. 2 and Table 2.

**Table 2:** Bias Coding for Included Studies

	Random sequence generation (Selection Bias)	Allocation concealment (Selection bias)	Blinding of participants and personnel (Performance bias)	Blinding of outcome assessment (Detection bias)	Incomplete outcome data (Attrition bias)	Selective reporting (Reporting bias)	Other sources of bias (Other bias)
Annesi (2002) [19]	Low	Low	Unclear	Low	Low	Low	Low
Calzolari et al. (2017) [20]	High	High	Unclear	Low	Low	High	Low
Carrera et al. (2017) [21]	Low	Low	Unclear	Low	Low	Low	Low
Carrera et al. (2018) [18]	Low	Low	Unclear	Low	Low	Low	Low
Courneya et al. (1997) [22]	Low	Low	Unclear	Low	Low	Low	Low
Estabrooks et al. (1996) [23]	Low	Low	Unclear	Low	Low	Low	Low
Marz (2017) [24]	Low	Low	Unclear	Low	High	Low	Low
Muller and Habla (2018) [17]	Low	Low	Unclear	Low	Low	Low	Low
Nigg et al. (1997)a [25]	Low	Low	Unclear	Low	Low	Low	Low
Nigg et al. (1997)b [26]	Low	Low	Unclear	Low	Low	Low	Low
Rohde et al. (2017) [27]	Low	Low	Unclear	Low	Low	Low	Low
Spangenberg (1997) [28]	Low	Low	Unclear	Low	Low	Low	Low
Thompson et al. (1980) [29]	Low	Low	Unclear	Low	Low	Low	Low
Wankel et al. (1977) [16]	Low	Low	Unclear	Low	Low	Low	Low

### Effectiveness

Since it was not appropriate to combine the results of the included studies into a meta-analysis due to the heterogeneity of the interventions, exploratory analyses were performed to assess the effects of each of the interventions. Effect sizes were calculated to analyse which of the interventions had the largest effect on attendance over the control group and are reported in Table 1. Only two studies, reporting four interventions, reported a large (*d* = 1.00, *d* = 1.37, *d* = 1.45) or medium effect size (*d* = 0.60) [16, 19]. All of the remaining studies reported small or trivial effect sizes. Of the remaining studies, the largest was an effect size of *d* = 0.38 [22] and the smallest was *d* = 0.004 [27].

### Behaviour change techniques

None of the studies explicitly reported the BCTs included in the interventions. Each intervention was therefore coded to identify BCTs in line with the BCT taxonomy (v1). Overall, 13 BCTs were coded across the 20 interventions. Four interventions included “Prompts/cues” (BCT 7.1) [17, 18, 20, 23]). Three studies reported “Incentive (outcome)” (BCT 10.8) [21, 22, 27]). “Pros and cons” (BCT 9.2) ([16, 26]) was reported by two studies. Each of the following BCTs were reported once: “Goal setting (behaviour)” (BCT 1.1) [19], “Problem solving” (BCT 1.2), [16], “Action planning” (BCT 1.4) [18] “Review behaviour goal(s)” (BCT 1.5) [19], “Feedback on behaviour” (BCT 2.2) [27], “Self-monitoring of behaviour” (BCT 2.3) [25], “Material incentive (behaviour)” (BCT 10.1) [24] and “Future punishment” (BCT 10.11) [24]. Two additional BCTs were included as additional codes as these were not identified within the BCT. These additional codes were identified once each: “Perceived choice” [29] and “Self-prophecy” [28] (Table 3).

**Table 3:** BCTs Included in the Interventions

	Effect Sizes	1.1: Goal Setting (behaviour)	1.2: Problem Solving	1.4: Action planning	1.5: Review behaviour goals(s)	2.2: Feedback on behaviour	2.3: Self-monitoring of behavior	7.1: Prompts/cues	9.2: Pros and cons	10.1: Material incentive (behavior)	10.8: Incentive (outcome)	10.11: Future punishment	Additional BCT: “Perceived choice”	Additional BCT: “Self-prophecy”
Annesi (2002) [19]	1.37	x			x									
Calzolari et al. (2017) [20]	0.16, 0.08							x						
Carrera et al. (2017) [21]	0.08										x			
Carrera et al. (2018) [18]	0.10			x				x						
Courneya et al. (1997) [22]	0.38										x			
Estabrooks et al. (1996) [23]	0.05							x						
Marz (2017) [24]	0.33, 0.23									x		x		
Muller and Habla (2018) [17]	0.01							x						
Nigg et al. (1997)a [25]	0.08, 0.20. 0.02						x							
Nigg et al. (1997)b [26]	0.31								x					
Rohde et al. (2017) [27]	0.03, 0.004					x					x			
Spangenberg (1997) [28]	0.18, 0.10													x
Thompson et al. (1980) [29]	0.29												x	
Wankel et al. (1997) [16]	1.45, 1.00, 0.60		x						x					

### Effectiveness of the BCTs

The study reporting the intervention with the largest effect size (*d* = 1.45) used “Pros/cons” and “Problem solving” [16]. This study also reported two additional interventions using “Pros/cons” and “Problem solving”; one with a large effect size (*d* = 1.00) and one with a medium effect size (*d* = 0.60). One other intervention had a large effect size (*d* = 1.37 [19]) using “Goal setting (behaviour)” and “Review behaviour goals”. These BCTs were not used in any of the other interventions. The BCTs used in interventions associated with small or trivial effect sizes were as follows: “Incentive (outcome)” (*d* = 0.004, *d* = 0.03, *d* = 0.08, *d* = 0.14, *d* = 0.29, *d* = 0.38); “Material incentive (behaviour)” (*d* = 0.04, *d* = 0.23, *d* = 0.33); Future punishment” (*d* = 0.04, *d* = 0.23, *d* = 0.33); “Pros and cons” (*d* = 0.31); “Perceived choice” (*d* = 0.29); “Self-prophecy” (*d* = 0.10, *d* = 0.18); “Prompts/cues” (*d* = 0.01, *d* = 0.05, *d* = 0.06, *d* = 0.10, *d* = 0.16); “Action Planning” (*d* = 0.10); “Self-monitoring of behaviour” (*d* = 0.08); and “Feedback on behaviour” (*d* = 0.004, *d* = 0.03).

## Discussion

### Main findings

The main aim of this systematic review was to understand the effectiveness of behaviour change interventions that aimed to increase attendance of members in health and fitness venues. Interventions with the largest effects on attendance utilised problem solving, pros/cons, goal setting (behaviour) and reviewing behaviour goals as behaviour change techniques (BCTs). Aside from one other intervention which had a medium effect size and also utilised problem solving and pros/cons, the remaining interventions had small or trivial effects on attendance behaviour. Given that only two studies (with combined sample size of 475) showed a moderate to large effect size, there is a limited evidence base from which to draw extensive conclusions on which BCTs could be effective in increasing attendances at health and fitness venues.

Pros/cons and problem solving showed the strongest evidence of effectiveness thereby demonstrating the potential utility of these techniques to increase attendances at health and fitness venues. The decisional balance of perceived advantages and disadvantages of change, such as pros/cons, is identified as one of three key factors that mediate behaviour change within the transtheoretical model of behaviour change (TTM) [30]. However, it should be noted that one intervention utilising pros/cons as the only BCT in the current review had a small effect (*d* = 0.31) [26]. The findings could be influenced by which BCTs pros/cons is paired with. Thus more research is therefore necessary to understand how this BCT can be most effectively applied to increase attendances at health and fitness venue.

The second highest effect sizes were found for interventions that included the BCTs goal-setting (behaviour) and review behaviour goals. These BCTs have been found to be effective techniques in a previous meta-analysis of PA interventions which found that interventions that combined goal setting along with self-monitoring [31] had the largest effect sizes. The meta-analysis also found that other behaviour change techniques derived from control theory [32], such as prompting intention, providing feedback on performance, and prompting review of goals were associated with larger effect sizes [31]. Interventions derived from control theory have also been found to be associated with greater changes in intention and stages of change in a review of how interventions can increase motivation for PA [33]. In the intervention included in the current review [19], members also met with a health and fitness professional every six weeks which suggests that face-to-face contact could be a good means through which to review behavioural goals.

The most common behaviour change technique, used in four studies, was prompts/cues [17, 18, 20, 23]. The second most common behaviour change technique, used in three studies, was financial incentives; however, the effects of financial incentives on attendance were small or trivial [21, 22, 27], although when financial incentives were framed as a ‘loss’ they had a stronger effect (*d* = 0.33) on attendance [24]. The behavioural economics literature has a wealth of research investigating the ‘loss aversion’ effect on individuals’ behaviour, notably that individuals tend to prefer avoiding losses than acquiring equivalent gains [34]. The majority of this research has been related to monetary gains and losses and how individuals respond to various decisions related to how much they could gain or lose in a specific situation. Further research is needed to understand how the users of health and fitness venues respond to the framing of financial losses and rewards to incentivise attendance.

The mode of delivery might also impact on intervention effectiveness. For example, the intervention with the highest effect size [16] was delivered via telephone and the second largest effect size [19] was delivered ‘face-to-face’ such that participants attended a number of pre-arranged 40 min sessions with a health and fitness professional. These methods of delivery were in contrast to many of the studies that had small or trivial effect sizes. In the two studies that had the smallest effect sizes ([22] [23];) participants had minimal face-to-face contact. For example, in both of these studies, the intervention conditions received the intervention in the post with instructions of what they needed to do. It could be that participants had low engagement with these interventions which may partially explain the trivial and non-significant effects. Similarly trivial effects were reported in other studies which had minimal face-to-face contact [20, 21]. One potential advantage of using methods not requiring personal contact is the high number of participants they can reach. However, these delivery methods may have lower effectiveness due to lower levels of participant engagement. Cost-effectiveness studies are therefore required to explore this trade-off between scale and engagement in interventions. Given the current findings it would also appear important to understand how interventions that have minimal direct contact with participants can be effective in increasing attendances.

### Implications of findings

This systematic review identified 14 studies reporting 20 interventions that sought to increase attendance in members at health and fitness venues. Of these, only three interventions showed a large effect. Given the results in the current review, interventions could include pros/cons alongside problem solving techniques and goal setting alongside reviewing behaviour goals to increase attendance in health and fitness venues. It is important to note that these findings were from only two separate studies; these implications should therefore be treated with caution. The inclusion of other BCTs taken from control theory, such as self-monitoring, should also be considered as they have been associated with large effect sizes in increasing motivation for PA [33]. There are also implications for the delivery of interventions. In particular, using a direct contact method of delivery may increase intervention effectiveness as it may lead to greater engagement than methods that do not directly interact with participants. Notably, the BCTs with the highest effect sizes were only reported in two studies. Although these could be effective in increasing attendances, additional research is required to replicate these findings. Apart from the use of four BCTs, other interventions included in the review had only small or trivial effects on attendance. More studies are needed to test a greater range of theory-based BCTs that have been found to be effective in other contexts. Identifying the BCTs that are best able to increase attendances at health and fitness venues may also help to increase PA at a population level given the large numbers of people who are members of such venues, but currently under-utilise them.

### Strengths and limitations of this review

The current review had a number of strengths. First, it is the first systematic review to evaluate the effectiveness of interventions to increase attendance in health and fitness venues. Second, the study reviewed studies that had electronically recorded attendance at the health and fitness venue. This measurement provides an objective assessment of attendance at venues and potential change as a result of interventions. Third, the utilisation of the behaviour change taxonomy also enabled a more detailed and systematic analysis of the likely active ingredients of successful interventions.

The current review also had some limitations. First, the conclusions are based on only 14 studies. More studies are therefore needed to identify the BCTs and components of interventions that could increase attendances at health and fitness venues. Second, none of the studies explicitly described the BCTs used within the study. The studies in the current review had to be coded to identify which BCTs had been included, often on the basis of limited information. Third, the studies included different monitoring periods which might have reduced the ability to compare effectiveness between interventions. However, there was no evidence that the length of the monitoring period was associated with larger or smaller effect sizes. Finally, the behaviour change technique taxonomy did not cover all of the BCTs identified.

## Conclusion

Overall, this systematic review has reported on the current evidence base on which BCTs can be effective in increasing attendance at health and fitness venues. Whilst the available evidence suggests utilising pros/cons alongside problem solving and goal-setting (behaviour) alongside reviewing behaviour goals may be effective, there are only a limited number of studies in this field. Small sample sizes and small effect sizes across the majority of interventions make it difficult to draw definitive conclusions and further studies are therefore required to provide greater certainty about which techniques BCTs are likely to increase attendances at health and fitness venues.

## Data Availability

All data generated or analysed during this study are included in this published article.

## Abbreviations

PA: Physical activity
PHE: Public Health England
